# Development of the forward parachute reaction and the age of walking in near term infants: a longitudinal observational study

**DOI:** 10.1186/1471-2431-9-13

**Published:** 2009-02-16

**Authors:** Domenico MM Romeo, Matteo Cioni, Mariacristina Scoto, Filippo Palermo, Alessandra Pizzardi, Anna Sorge, Mario G Romeo

**Affiliations:** 1 Division of Child Neurology and Psychiatry, Department of Paediatrics, University of Catania, Catania, Italy; 2 Laboratory of Gait and Motion Analysis, Chair of Physical Medicine & Rehabilitation, School of Medicine, University of Catania, Catania, Italy; 3 Department of Internal Medicine, University of Catania, Catania, Italy; 4 Department of Paediatrics, University of Catania, Catania, Italy; 5 Neonatal Intensive Care Unit, Department of Paediatrics, University of Catania, Catania, Italy

## Abstract

**Background:**

Near term infants are a main part of preterms. They are at higher risk for mortality and morbidity than term infants and could show a quite different development of tone and reflexes from them. The aim of the present study was to describe longitudinally, in a large sample of healthy near term infants, the development of the forward parachute reaction (FPR) and its correlation with the age of acquisition of independent walking.

**Methods:**

The assessment of FPR (as absent, incomplete or complete) was performed at 3, 6, 9, 12 months of corrected age in 484 infants, with a gestational age between 35.0 and 36.9 weeks. The age of acquisition of independent walking was monitored until its appearance. A correlation analysis was done between the age of walking and the acquisition of a complete or incomplete FPR, using the Spearman Rank correlation. The Mann-Withney U test was used to identify significant gestational age differences for the age of FPR appearance.

**Results:**

Most of infants had a two-step development pattern. In fact, they showed at first an incomplete and then a complete FPR, which was observed more frequently at 9 months. An incomplete FPR only, without a successive maturation to a complete FPR, was present in the 21% of the whole sample. Infants with a complete FPR walked at a median age of 13 months, whereas those with an incomplete FPR only walked at a median age of 14 months.

**Conclusion:**

We identified two groups within our sample of near term infants. The first group showed a progressive maturation of FPR, whereas the second one was characterised by the inability to get a complete pattern, within the one year observation's period. Furthermore, we observed a trend toward a delayed acquisition of independent walking in the latter group of infants.

## Background

During the last two decades, the population of near term infants (34 weeks through 36 weeks of gestation) is increased progressively and it is actually the 70% of the whole population of preterms [1-3]. Improvement of medical interventions during pregnancy and the development of Neonatal Intensive Care Units, are considered the main causes of this phenomenon [1,3,4]. This population is reported to be at higher risk for mortality and morbidity than term infants [1,5]. In despite of this observation, near term infants are not usually included in follow-up programs [1]. We have previously observed [6] by a structured neurological examination, that healthy near term infants, during the first year of life, could show lower scoring for tone and reflexes than term infants. Consequently, we have suggested that during this period, these infants follow a peculiar slope of neuromotor development [6].

The forward parachute reaction (FPR) is a milestone "key" for the neuromotor development of infants. In fact, several authors showed that infants need a previous acquisition of this reaction before standing and walking [7,8]. In clinical practice, the FPR is evoked by suspending the infant ventrally and by dropping him suddenly with the head directed towards a surface [7-10]. We can observe both a complete or incomplete FPR. The former consists in a symmetric extension of arms with spreading of fingers on a quick approach to a visual surface [7,10], whereas the latter is characterised by a light forward extension of arms, with a partial spreading of fingers [7,10]. In term born infants, some authors [7,8,10] have shown that a complete FPR is developed within 12 months of age, whereas we have less information about the timing of the incomplete one [7]. In preterm infants, one study only [9] investigated the FPR, in a small group of infants, with findings similar to term born babies, but information is still lacking regarding near term infants. Then, the aim of this study was to describe, in a large sample of near term infants, both the characteristics and the timing of FPR throughout the first year of life and its correlation with the age of acquisition of independent walking.

## Methods

The infants described in this study are a part of a large cohort admitted to the Neonatal Unit of the Department of Paediatrics of the University of Catania between January 2001 – December 2005. They were consecutively enrolled in a follow-up research program until the age of 2 years. Although the near term well babies are not likely to stay at hospital longer than a few days, our follow-up research program included one assessment for cranial ultrasound (US) during the first week, one after 14 days and one at term age. The last two US scans were done after discharge from the hospital. As a part of the follow up program, neurological development was systematically followed with a structured neurological assessment, performed at 3, 6, 9 and 12 months of corrected age (CA) [11], and a neurodevelopmental assessment at 2 years CA including the Touwen's neurological examination [7] and the Clinical Adaptive Test/Clinical, Linguistic and Auditory Milestone Scale (CAT-CLAMS) [12]. The inclusion criteria of the present study were: i) a gestational age (GA) between 35.0 and 36.9 weeks, ii) a normal cranial US or transient flares (lasting less than 2 weeks) or germinal layer haemorrhages grade 1 (IVH 1) according to Volpe [13], iii) a normal outcome at 2 years CA defined as a normal neurological examination and a CAT-CLAMS quotient above 85, iv) a body weight within the 10° and 90° centile during the first year of age. The exclusion criteria were the presence of small for gestational age (weight below the 10^th ^percentile), major congenital malformations, severe postnatal infectious diseases, metabolic or haematological complications and variable follow-up program. The study protocol was previously approved by the Ethics Committee of our Institution and a signed informed consent was obtained from parents.

### Assessment of Forward Parachute Reaction

The FPR was assessed at 3, 6, 9, 12 months CA in all infants included in the present study.

FPR was evoked by holding the infant by the hips in a prone position (initial position) and by dropping him suddenly downwards at 45 degrees with the head directed towards a surface with the eyes open. The manoeuvre was applied when the infant was in the most appropriate behavioural state, corresponding to state 4 of Brazelton (alert – bright eyed – focused attention minimal motor activity) [14]. It was repeated two times with a reproducibility of 100%. According to Touwen [7] a score of 0 was given for an absent reaction, a score of 1 when it was incomplete (slight forward extension of the arms with or without spreading of the fingers) and a score of 2 for a complete reaction (symmetric extension of arms with extension and spreading of fingers). The assessment of FPR was performed by one of the two examiners (DMMR, MS). The inter-observer reliability was performed in 100 infants by the two examiners with an inter-observer correlation coefficient > 0.90.

### Independent walking onset

The age of onset of independent walking was defined as the time when the infant took the first five successive steps without support [15]. The parents were instructed to note the exact date when the infant began walking independently and phone calls were made after 12 months, to ensure accurate documentation of walking.

### Statistical analysis

Values are reported as median, minimum-maximum and 25°–75° percentiles. For analysis purposes, infants were divided in two groups. A first group (group A) was composed by infants who gained a complete FPR within the observation's period; in this group are enclosed those infants showing an incomplete FPR followed by a complete FPR too. A second group (group B) was represented by those who had an incomplete FPR only. A third group of infants contemplate those infants with no FPR (both incomplete or complete) within the first year of life; because of the limited number of this group, it was not enclosed in the statistical analysis. The number of infants who had a complete or incomplete FPR at 3, 6, 9, 12 months were calculated. A correlation analysis was done between the age of walking and the acquisition of a complete or incomplete FPR, using the Spearman Rank correlation. The Mann-Withney U test was used to identify significant GA differences for the age of FPR appearance. The level of significance was set at p < 0.05.

## Results

During the study period, 648 consecutive newborns with a GA between 35,0 and 36,9 weeks were discharged from our Neonatal Unit; from this population 164 babies were excluded because of major lesions at US scanning (n = 86) or an incomplete follow-up program (n = 78); 484 infants fulfilled the inclusion criteria; 228 were born at 35 weeks and 256 at 36 weeks GA. A Normal cranial US was found in 415 infants, whereas transient flares or IVH 1 were respectively observed in 59 and 10 infants. The 51% of this sample was male. No significant differences (p > 0.05) between infants born at 35 and those born at 36 weeks were found for the age of acquisition of FPR. Infants with minor lesions on US showed similar findings to those observed in the group with normal US. The results of neuromotor assessments performed during the first year of life are reported in a previous paper [6]. All the infants reported a normal neurodevelopmental evaluation with a developmental quotient > 85. The two groups of infants (Group A and B) reported similar findings in both the neuromotor evaluation, during the first year of life, and the neurodevelopmental assessment at two years.

### Forward parachute reaction

**Figure 1:**
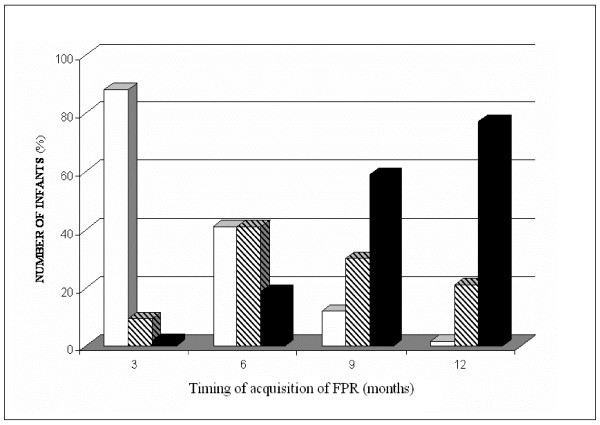
**Different typology of forward parachute reactions (FPR) and number of near term infants (%) showing each type of FPR, at the four evaluations during the first year of age (black = complete FPR; stripped = incomplete FPR; white = absent FPR)**.

In Fig. 1, at 3 months a limited number of infants showed a FPR which was complete in the 2% and incomplete in 10% of them. At 6, 9, 12 months, we observed a progressive development of a complete FPR, respectively in the 19%, 59% and 77% of infants. By contrast, an incomplete FPR was identified more frequently at 6 months (41%) than at 9 (30%) and 12 months (21%). Eight infants (2%) did not show any FPR during our observation's period.

**Figure 2:**
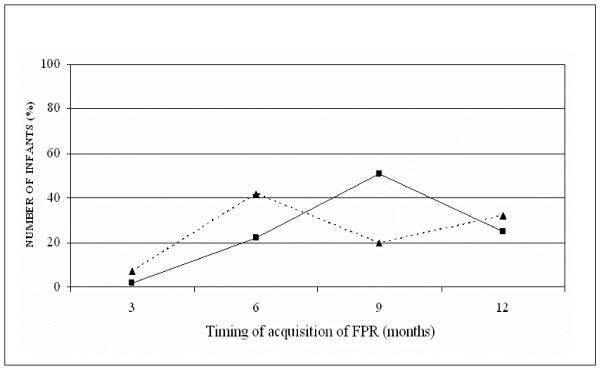
**Number of infants (reported as percent of the total number within each group) showing a complete (group A) or an incomplete (group B) FPR, during the first year of age**. Group A = Black square; Group B = Black triangle.

Fig. 2 shows the progressive appearance of FPR, both complete (Group A) and incomplete (Group B), as monitored at different ages of life (3, 6, 9, 12 months). It can be seen that the slopes peak differently. In fact, most of infants of the group A showed a complete FPR at 9 months, whereas the majority of group B showed an incomplete FPR at 6 months.

### Independent walking

**Figure 3:**
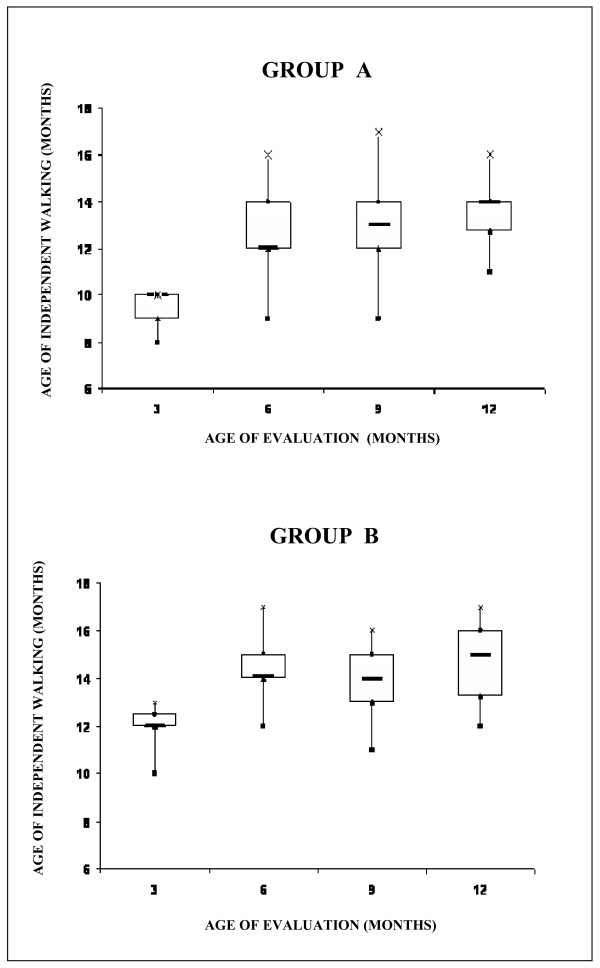
**Ages of acquisition of independent walking in the groups of infants showing respectively a complete (group A) or an incomplete FPR (group B)**. Scatter plots show Min (Black square), Max (x), 25^th ^centiles (Black triangle), 75^th ^centiles (Black circle) and median (Black line) values.

All infants showed an independent walking within 17 months of age, with a median of 13 months. Infants with a complete FPR at 3 months developed precociously an independent walking at a median age of 10 months, whereas those who had a complete FPR at 6, 9 and 12 months walked respectively at a median age of 12, 13 and 14 months (Fig. 3, group A). By contrast, for those infants showing an incomplete FPR only at 3 months, the median age of acquisition of an independent walking was 12 months, whereas it was of 14 months for those infants with an incomplete FPR at 6 and 9 months; those with an incomplete FPR at 12 months, gained an independent walking further delayed up to a median of 15 months (Fig. 3, group B). Those infants who did not show neither a complete nor an incomplete FPR walked definitively later between 15 and 17 months (median 15 months).

The correlation analysis (r) between the age of acquisition of an independent walking and the age of a complete FPR by 12 month was 0.32 (p < 0.0001) whereas for those with an incomplete FPR only, *r *was 0.24 (p < 0.05).

## Discussion

Our study investigated the development of FPR in a large sample of near term infants, during the first year of life. We found that most of infants showed a complete FPR as previously shown both in term [10] and preterm infants [9] which was correlated to the age of acquisition of independent walking. Furthermore, we identified another consistent group of infants (the 21% of the whole sample) who developed an incomplete FPR only, in most cases at 6 months, and acquired an independent walking later on.

The possible physiological background of FPR was proposed by Touwen [7], who suggested that it could depend on the influence of sensory inputs (visual and vestibular). Its functional significance could be related to the need to protect the infant from injuries as suggested by Milani-Comparetti [8]. The timing of FPR development in healthy term infants has been studied by Touwen [7] who showed a complete FPR after 6 months of age. In the same line of evidence, Wenzel [10] demonstrated that FPR is fully developed at about 9 months. More recently, Ohlweiler et al [9] studied the development of FPR in a group of 40 healthy preterm infants (< 30 – 36.6 weeks) and pointed out that most of them had a FPR at 9 months. The comparison of our data with those reported in the literature [9,10] confirms that most infants had a complete FPR at 9 months, even in a population of near term infants, with a longitudinal trend similar to that of term infants [10].

The incomplete FPR is characterised by an incomplete extension and/or by an asymmetric extension of the upper limbs and/or of closed hands [7,10]. Its functional significance is not still clarified, but it is probable that it is the expression of an immature pattern of sensory-motor development. Touwen [7] firstly found the presence of an incomplete FPR in a group of 51 healthy term infants. Its appearance was evident since the age of 3 months and was still present at the age of 12 months, but this author did not specify the number or percent of infants showing an incomplete FPR. In the same line of evidence, Frisone et al. [16] showed that 1/3 of their group of preterm infants (< 32 weeks) had an incomplete/absent FPR when assessed between 9 and 18 months. In keeping with these previous investigations [7,10,16], our results indicate that FPR could be incompletely evoked, without a successive maturation in a complete one, during the first year of age. Since, the assessment of FPR was limited to this period, it is possible that the maturation of FPR was completed after 12 months.

An interesting result of our study is the presence of a small subgroup of infants who develop an early incomplete reaction (at 3 months) showing an independent walking around 12 months. These infants were not able to gain a complete FPR within the first year of life and, in the same line of evidence reported by other authors [7,8], it seems improbable that they could have developed a complete FPR at an older age, after the acquisition of an independent walking. This suggests that the incomplete FPR can be found as a transient pattern in infants who develop a complete FPR within 12 months, but also as a specific type of FPR in normal infants who walks around 12 months and probably who never will develop a full reaction.

The systematic observation of the sensory-motor development of infants has shown that it occurs according to a timed calendar. In fact, a normal infant acquires the sitting position at about 6 months, on all-fours at 9 months, supported walking at 12 months and independent walking between 12–15 months [7,8,17,18]. This phenomenon is the expression of a progressive brain maturation and namely of antigravity neural systems and locomotor spinal centers [19,20]. In order to get a first step, an infant basically needs parachute reactions, stability in standing and stepping abilities. The absence or the inefficiency of one of these patterns can delay the process of acquisition of an independent walking [18-20]. Interestingly, Jaffe et al. [21] showed that in a sample of 190 term infants the independent walking did not occur without a previous acquisition of parachute reactions and that only 2 infants walked without those reactions. Successively, the same authors [22] reported that a delayed appearance of FPR was related to a late acquisition of independent walking. In the light of this consideration, the timing of development of FPR has an important role in the ontogenetic process of infants. In our study, an independent walking at 8–10 months was attained by a small number of infants who showed a complete FPR at 3 months, indicating that their global motor development and maturation of sensory inputs were the most precocious in that infant's group. The age of walking of our near term infants with complete FPR was similar to that of those term infants studied by Jaffe et al. [21] (both at about 13 months), whereas those who showed an incomplete FPR developed an independent walking later at a median age of 14 months. On the other hand, infants who did not develop a FPR, within the observation's period, walked at a median age of 15 months.

Then, our study shows for the first time not only the features and the development of FPR in near term infants, but also the temporal relationships between the development of an incomplete FPR and an independent walking.

A peculiar characteristic of our sample is represented by the high number of infants who were recruited for the study. In fact, it seems to be sufficiently consistent, from a statistical point of view, to follow the development of FPR during the first year of age. Since, the infants enrolled for this investigation were near term babies only, it is not possible to extend our observations to healthy infants born at term age. However, we can conclude by indicating that, in our near term sample, a milestone "key", as a complete FPR, follows a development quite overlapping to that of term infants and similarly correlated to the acquisition of independent walking. On the other hand, we should consider two main limitations of this study. Firstly, the fact that our observation's period was limited to the first year of age and secondly that the interval between each evaluation was of 3 months. Consequently, we cannot indicate the exact time of acquisition and maturation of FPR.

## Conclusion

In conclusion, by this study we point out the role played by the FPR in the process of near term infant's maturation and its correlation with the acquisition of functional abilities as independent walking. The knowledge of the maturation of these patterns could be useful for clinicians to identify those infants with motor delay. Future studies could be needed to enrol large samples of infants at low and high risk to identify the predictive value of FPR maturation in relation to the development of posture and movement.

## Abbreviations

FPR: Forward Parachute Reaction; US: Ultrasound; CA: Corrected Age; CAT-CLAMS: Clinical Adaptive Test/Clinical, Linguistic and Auditory Milestone Scale; GA: Gestational Age; IVH 1: Germinal layer haemorrhages grade 1; r: Correlation analysis.

## Competing interests

The authors declare that they have no competing interests.

## Authors' contributions

DMMR participated in the design of the study, performed the clinical neurological assessments and helped to draft the manuscript. MC participated in the design of the study and drafted the manuscript. MS performed the clinical neurological assessments. FP performed the statistical analysis. AP and AS participated in the design of the study. MGR conceived the study, participated in its design and coordination. All authors read and approved the final manuscript.

## Pre-publication history

The pre-publication history for this paper can be accessed here:



## References

[B1] Engle WA, Tomashek KM, Wallman C, the Committee on Fetus and Newborn (2007). Late-preterm infants: a population at risk. Pediatrics.

[B2] Shapiro-Mendoza CK, Tomashek KM, Kotelchuck M, Barfield W, Weiss J, Evans S (2006). Risk factors for neonatal morbidity and mortality among healthy late preterm newborns. Semin Perinatol.

[B3] Branum AM, Schoendorf K (2002). Changing patterns of low birth weight and preterm death in the United States, 1981–98. Paediatr Perinat Epidemiol.

[B4] Villar J, Abalos E, Carroli G, Giordano D, Wojdyla D, Piaggio G, Campodonico L, Gülmezoglu M, Lumbiganon P, Bergsjø P, Ba'aqeel H, Farnot U, Bakketeig L, Al-Mazrou Y, Kramer M, World Health Organization Antenatal Care Trial Research Group (2004). Heterogeneity of perinatal outcomes in the preterm delivery syndrome. Obstet Gynecol.

[B5] Escobar GJ, Clark RH, Greene JD (2006). Short-term outcomes of infants born at 35 and 36 weeks gestation: we need to ask more questions. Semin Perinatol.

[B6] Romeo DM, Cioni M, Guzzetta A, Scoto M, Conversano M, Palermo F, Romeo MG, Mercuri E (2007). Application of a scorable neurologic examination to near-term infants: longitudinal data. Neuropediatrics.

[B7] Touwen B (1976). Neurological Development in Infancy. Clinics in Developmental Medicine No 58.

[B8] Milani-Comparetti A, Gidoni EA (1967). Routine development examination in normal and retarded children. Dev Med Child Neurol.

[B9] Ohlweiler L, da Silva AR, Rotta NT (2002). Parachute and lateral propping reactions in preterm children. Arq Neuropsiquiatr.

[B10] Wenzel D (1978). The development of the parachute reaction: a visuo-vestibular response. Neuropediatrie.

[B11] Haataja L, Mercuri E, Regev R, Cowan F, Rutherford M, Dubowitz V, Dubowitz L (1999). Optimality score for the neurologic examination of the infant at 12 and 18 months of age. J Pediatr.

[B12] Capute AJ, Accardo PJ, Capute AJ, Accardo PJ (1991). Language assessment. Developmental disabilities in infancy and childhood.

[B13] Volpe JJ (2001). Neurology of the Newborn.

[B14] Brazelton TB, Nugent JK (1995). Neonatal Behavioral Assessment Scale.

[B15] Jeng SF, Yau KI, Liao HF, Chen LC, Chen PS (2000). Prognostic factors for walking attainment in very low birth weight preterm infants. Early Hum Dev.

[B16] Frisone MF, Mercuri E, Laroche S, Foglia C, Maalouf E, Haataja L, Cowan F, Dubowitz L (2002). Prognostic value of the neurologic optimality score at 9 and 18 months in preterm infants born before 32 weeks gestation. J Pediatr.

[B17] Amiel-Tison C, Grenier A (1983). Neurologic evaluation of the newborn and the infant.

[B18] Woollacott MH, Shumway-Cook A (1990). Changes in posture control across the life span-A Systems Approach. Phys Ther.

[B19] Leonard CT, Hirschfeld H, Forssberg H (1991). The development of independent walking in children with cerebral palsy. Dev Med Child Neurol.

[B20] Forssberg H (1985). Ontogeny of human locomotor control. I. Infant stepping, supported locomotion and transition to independent locomotion. Exp Brain Res.

[B21] Jaffe M, Kugelman A, Tirosh E, Cohen A, Tal Y (1994). Relationship between the parachute reactions and walking in normal infants. Ped Neurol.

[B22] Jaffe M, Tirosh E, Kessel A, Kugelman A, Tal Y (1996). The parachute reactions in normal and late walkers. Ped Neurol.

